# Treatment of Cystitis by Atropia Enemata

**Published:** 1876-08

**Authors:** 


Treatment of Cystitis by Atropia Enemata.—Dr. G. W. Semple (*Virginia Med. Monthly*) recommends in strong terms, as an almost unfailing remedy in "acute cystitis" occurring in young girls and in women about the change of life, the injection into the rectum of a solution of atropia. He directs the injection of from 40 to 60 minims of a solution of sulphate of atropia, one grain to eight ounces of water, to which is added sufficient carbolic acid to prevent the formation of organic matter and the deposit of atropia. The dose is added to ℥ss of water for administration, and given twice in twenty-four hours. It uniformly and immediately arrests the frequent strangury and painful micturition, gradually checks the mucous and sanguineous discharges, and relieves the subrapubic pain with the cystic inflammation. When the urine is alkaline, nitro-muriatic acid is given to correct it; and when it is so acid as to irritate, the acidity is corrected by antacid remedies, of which the bicarbonate of potash, with subnitrate of bismuth, is generally preferred, because of the tonic effect of the bismuth and its very soothing effect on the mucous surfaces of the urinary organs. When constipation exists, which is frequent, it is relieved as occasion requires, generally by the German pulvis glycirrhizæ compositus, until the bowels begin to act regularly from the effect of the atropia, which generally soon results.—*Pacific Med. and Surg. Journal.*

